# Fatigue-induced changes in knee-extensor torque complexity and muscle metabolic rate are dependent on joint angle

**DOI:** 10.1007/s00421-021-04779-1

**Published:** 2021-08-05

**Authors:** Jamie Pethick, Samantha L. Winter, Mark Burnley

**Affiliations:** 1 School of Sport, Rehabilitation and Exercise Sciences, University of Essex, Wivenhoe Park, Colchester,, CO4 3WA UK; 2 School of Sport, Exercise and Health Sciences, Loughborough University, Loughborough, UK; 3 Endurance Research Group, School of Sport and Exercise Sciences, University of Kent, Canterbury, Kent UK

**Keywords:** Exercise, Oxygen consumption, Non-linear dynamics, Complexity endurance, Muscle fatigue

## Abstract

**Purpose:**

Joint angle is a significant determinant of neuromuscular and metabolic function. We tested the hypothesis that previously reported correlations between knee-extensor torque complexity and metabolic rate (${\text{m}\dot{\text{V}}\text{O}}_{{2}}$) would be conserved at reduced joint angles (i.e. shorter muscle lengths).

**Methods:**

Eleven participants performed intermittent isometric knee-extensor contractions at 50% maximum voluntary torque for 30 min or until task failure (whichever occurred sooner) at joint angles of 30º, 60º and 90º of flexion (0º = extension). Torque and surface EMG were sampled continuously. Complexity and fractal scaling of torque were quantified using approximate entropy (ApEn) and detrended fluctuation analysis (DFA) α. ${\text{m}\dot{\text{V}}\text{O}}_{{2}}$ was determined using near-infrared spectroscopy.

**Results:**

Time to task failure/end increased as joint angle decreased (*P* < 0.001). Over time, complexity decreased at 90º and 60º (decreased ApEn, increased DFA *α*, both *P* < 0.001), but not 30º. ${\text{m}\dot{\text{V}}\text{O}}_{{2}}$ increased at all joint angles (*P* < 0.001), though the magnitude of this increase was lower at 30º compared to 60º and 90º (both *P* < 0.01). There were significant correlations between torque complexity and ${\text{m}\dot{\text{V}}\text{O}}_{{2}}$ at 90º (ApEn, *r* =  − 0.60, *P* = 0.049) and 60º (ApEn, *r* =  − 0.64, *P* = 0.035; DFA *α*, *ρ* = 0.68, *P* = 0.015).

**Conclusion:**

The lack of correlation between ${\text{m}\dot{\text{V}}\text{O}}_{{2}}$ and complexity at 30º was likely due to low relative task demands, given the similar kinetics of ${\text{m}\dot{\text{V}}\text{O}}_{{2}}$ and torque complexity. An inverse correlation between ${\text{m}\dot{\text{V}}\text{O}}_{{2}}$ and knee-extensor torque complexity occurs during high-intensity contractions at intermediate, but not short, muscle lengths.

## Introduction

The relationship between muscle length (or joint angle) and torque has been extensively studied and described (Gordon et al. 1966; Lanza et al. 2017). In the knee extensors, for example, as the knee joint is moved into flexion, the muscles of the quadriceps femoris increase in length and maximal torque increases in a parabolic fashion up to approximately 75° (with 0° being full extension), before decreasing with further increases in length (Rassier et al. 1999; Becker and Awiszus 2001). Joint angle does not, however, simply affect the ability to produce maximal torque; rather, it is also a critical factor in determining endurance and neuromuscular fatigue mechanisms (Fitch and McComas 1985; Kooistra et al. 2006) and torque fluctuations (Ofori et al. 2018).

Endurance during submaximal isometric contractions in a variety of muscle groups has been demonstrated to be greater at more extended joint angles (Fitch and McComas 1985; McKenzie and Gandevia 1987; Ng et al. 1994). In the knee extensors, short muscle lengths and lower angles of flexion (e.g. ~ 30°) are associated with slower rates of fatigue development than the optimal torque production angle (~ 75°) and the greater angles of flexion (i.e. 90°) typically used in physiological testing, even at the same relative contraction intensity (Kooistra et al. 2005; Place et al. 2005). Unlike the muscle length–torque relationship, which can be largely (though not exclusively) explained using the sliding filament theory (Gordon et al. 1966; Lanza et al. 2017), the mechanism(s) for the muscle length–endurance relationship is less obvious. Numerous mechanisms have been proposed, with differences in central activation and blood flow seemingly discounted (Kooistra et al. 2005; Place et al. 2005). It has been demonstrated that muscle metabolic rate (measured using near-infrared spectroscopy [NIRS] and the rates of concentration change in oxyhaemoglobin and deoxyhaemoglobin) in the knee extensors is significantly lower, and the rate of increase in metabolic rate during fatiguing contractions is significantly slower, at 30° of flexion compared to 60° and 90° (Hisaeda et al. 2001; De Ruiter et al. 2005; Kooistra et al. 2006). These results suggest that joint angle-related differences in metabolic cost may be responsible for the differences in endurance seen at different joint angles.

Muscle torque output is characterised by constant inherent fluctuations (Slifkin and Newell 1999; Enoka et al. 2003). Such fluctuations in muscular output are of functional significance, influencing the capacity to achieve a desired force and produce an intended movement trajectory (Enoka et al. 2003). Traditionally, these fluctuations have been quantified according to their *magnitude*, using measures such as the standard deviation (SD) and coefficient of variation (CV). Recently, fluctuations in muscular output have also started to be quantified according to their temporal *structure*, or “complexity” (Slifkin and Newell 1999). Complexity measures quantify the degree of signal irregularity (e.g. approximate entropy, ApEn; Pincus 1991) and identify the presence of long-range fractal correlations (e.g. detrended fluctuation analysis, DFA; Peng et al. 1994). Importantly, these are properties that magnitude-based measures of fluctuations cannot quantify (Goldberger et al. 2002a). Thus, complexity measures provide additional insight into torque control beyond that provided by traditional magnitude-based measures. The presence of a complex output is believed to be a marker of system adaptability (Goldberger et al. 2002a) and, in the context of muscle torque, reflects the ability to adapt motor output rapidly and accurately in response to task demands (Vaillancourt and Newell 2003).

Joint angle is a critical, though under-investigated, factor in determining the dynamics of muscle torque fluctuations (Ofori et al. 2018). Previous research on the magnitude of fluctuations has demonstrated a lower SD of fluctuations at more extended joint angles (Sosnoff et al. 2009; Ofori et al. 2018), though no difference between the linear slopes fitted to the SD–contraction intensity relationship between extended and flexed joint angles (Shinohara et al. 2006). Only one study to date has investigated muscle torque complexity at different joint angles (Ofori et al. 2018), indicating that more extended joint angles are associated with greater complexity. Moreover, the shape of the relationship between complexity and contraction intensity differed with joint angle; at a flexed angle (100°), there was no relationship between contraction intensity and ApEn, whilst at an extended angle (40°), there was a quadratic trend, taking the form of a shallow *U*-shape. These findings contrast with the linear relationship previously observed during knee extension contractions performed at 90° of flexion (Pethick et al. 2016, 2020). The mediating effect of joint angle on the relationship between contraction intensity and complexity therefore requires further work.

It has been suggested that physiological complexity should decrease as metabolic rate is increased (Seely and Macklem 2012). This is of interest in the present context, because muscle oxygen consumption (${\text{m}\dot{\text{V}}\text{O}}_{{2}}$) has also been shown to depend upon knee joint angle, with smaller ${\text{m}\dot{\text{V}}\text{O}}_{{2}}$ responses observed as the knee joint is extended (Kooistra et al. 2006). Our recent work (Pethick et al. 2019) has demonstrated that a fatigue-induced loss of complexity (measured using ApEn and the DFA α scaling exponent; Pethick et al. 2015, 2016) exhibited a modest negative correlation with the fatigue-induced increase in muscle metabolic rate (measured using NIRS during arterial occlusion), providing support for the hypothesised relationship between system complexity and metabolic rate. That muscle metabolic rate appears to be lower (Kooistra et al. 2006) and complexity greater (Ofori et al. 2018) at more extended joint angles appears to support Seely and Macklem’s (2012) hypothesis, as decreasing the relative demand on a system should predictably increase system complexity. However, no study yet has measured muscle metabolic rate and torque complexity simultaneously at different joint angles.

The purpose of the present study was to experimentally manipulate knee-extensor joint angle to determine whether the previously observed inverse relationship between knee-extensor torque complexity and metabolic rate (Pethick et al. 2019) is conserved at different joint angles, and therefore muscle lengths. In doing so, we also aimed to further investigate whether joint angle-dependent changes in metabolic rate are responsible for the greater endurance observed at shorter muscle lengths. Our secondary aim was to investigate the relationships between muscle torque complexity, contraction intensity and joint angle. The experimental hypotheses tested were: (1) that there would be an inverse correlation between muscle metabolic rate and muscle torque complexity at all joint angles tested; (2) that more extended joint angles (i.e. 30° of flexion) would slow the fatigue-induced increase in muscle metabolic rate (quantified using NIRS) compared to more flexed joint angles (i.e. 60º or 90º); (3) that more extended joint angles would slow the fatigue-induced reduction in muscle torque complexity (quantified by a slower rate of decrease in ApEn and a slower rate of increase in DFA *α*); and (4) that a linear relationship between muscle torque complexity and contraction intensity would be observed at all joint angles, with more extended joint angles associated with greater complexity (i.e. greater values of ApEn and lower values of DFA *α*).

## Materials and methods

### Participants

Eleven healthy participants (nine males, two females; mean ± SD: age 26.3 ± 6.0 years; height 1.75 ± 0.08 m; body mass 68.7 ± 9.7 kg) provided written informed consent to participate in the study, which was approved by the ethics committee of the University of Kent (Prop 122_2016_17), and which adhered to the Declaration of Helsinki (except for the inclusion of the protocol in a publicly accessible database). Participants were instructed to arrive at the laboratory in a rested state (having performed no strenuous exercise in the preceding 24 h) and to have consumed neither any food nor caffeinated beverages in the 3 h prior to arrival. Participants visited the laboratory at the same time of day (± 2 h).

### Experimental design

Participants visited the laboratory on four occasions, with a minimum of 48 h between each visit. During their first visit, participants were familiarised with all testing equipment and procedures, and the settings for the dynamometer and stimulator were recorded. During the next three visits, participants performed, in a randomised order, intermittent isometric contractions at 30°, 60° and 90° of knee flexion (short, medium and long muscle lengths, respectively) to task failure or for 30 min, whichever occurred sooner. In each trial, torque output was sampled continuously to allow the quantification of complexity, muscle activity was measured from the vastus lateralis and vastus medialis electromyogram (EMG), knee-extensor metabolic rate was assessed using near-infrared spectroscopy (NIRS) and arterial occlusion, and maximal voluntary contractions (MVCs) with supramaximal femoral nerve stimulation were used to quantify global (i.e. loss of maximal voluntary torque), central and peripheral fatigue.

### Dynamometry

During all visits, participants sat in the chair of a Cybex isokinetic dynamometer (HUMAC Norm; CSMi, Massachusetts, USA), initialised and calibrated according to the manufacturer’s instructions. Their right leg was attached to the lever arm of the dynamometer, with the seating position adjusted to ensure that the lateral epicondyle of the femur was in line with the axis of rotation of the lever arm. Participants sat with a relative hip angle of 85° and a relative knee angle of either 30°, 60° or 90°, with full extension being 0°. The lower leg was securely attached to the lever arm above the malleoli with a padded Velcro strap, whilst straps secured firmly across both shoulders and the waist prevented any extraneous movement and the use of the hip extensors during the isometric contractions. The seating position for each joint angle was recorded during the first visit and replicated during each subsequent visit.

### Femoral nerve stimulation

Electrical stimulation of the femoral nerve was used to assess neuromuscular fatigue processes, as described previously in Pethick et al. (2015). A carbon rubber electrode with adhesive gel (100 × 50 mm; Phoenix Healthcare Products Ltd., Nottingham, UK) acted as the anode and was placed lateral to the ischial tuberosity, on the posterior aspect of the leg. The position of the cathode was determined using a motor point pen (Compex; DJO Global, Guildford, UK), and based on the location in the femoral triangle giving the largest twitch and greatest peak-to-peak amplitude of the compound muscle action potential (M-wave) following single stimulation at 100 mA, using a constant-current variable voltage stimulator (Digitimer, DS7AH, Welwyn Garden City, UK). Following determination of the precise cathode location, an Ag/AgCl electrode coated in conductive gel (32 × 32 mm; Nessler Medizintechnik, Innsbruck, Austria) was placed over the femoral nerve.

The appropriate stimulator current was then established by incrementally increasing the current, in steps of 20 mA, until knee extensor torque and the M-wave response to single twitches had plateaued. This was confirmed with stimulation delivered during a contraction at 50% MVC to ensure that a maximal M-wave during an isometric contraction was also evident. Once this was obtained, the stimulator current was increased to 130% of the current producing a maximal M-wave. In all subsequent trials, doublet stimulation (two 200 µs pulses with 10 ms interpulse interval) was used.

### Surface EMG

The EMG of the vastus lateralis and vastus medialis were sampled using Ag/AgCl electrodes (32 × 32 mm; Nessler Medizintechnik, Innsbruck, Austria). Prior to attachment of the electrodes, the skin of the participants was shaved, abraded and cleaned with an alcohol swab over the belly of the muscle to reduce impedance. The electrodes were placed on the prepared skin over the belly of the muscle, parallel to the approximate alignment of the muscle fibres. A reference electrode was placed on prepared skin medial to the tibial tuberosity. The raw EMG signals were sampled at 1 kHz, amplified (gain 1000; Biopac MP150; Biopac Systems Inc., California, USA) and band-pass filtered (10–500 Hz; Biopac MP150; Biopac Systems Inc., California, USA).

### Muscle oxygen consumption

Muscle oxygen consumption (${\text{m}\dot{\text{V}}\text{O}}_{{2}}$) from the vastus lateralis was obtained using a continuous-wave NIRS device (Oxymon Mk III; Artinis Medical Systems, The Netherlands), calibrated according to the manufacturer’s instructions before each trial. The NIRS device generated light at three wavelengths (905, 850 and 770 nm) corresponding to the absorption wavelengths of oxyhaemoglobin (O_2_Hb) and deoxyhaemoglobin (HHb). An area at the level of the largest circumference of the vastus lateralis was shaved, abraded and cleaned with an alcohol swab. The NIRS optode was then placed at this location and secured with Velcro straps and biadhesive tape, such that the optode could not move during contractions. A blood pressure cuff (Hokanson E20 cuff inflator; D.E. Hokanson Inc., Bellevue, USA) was placed proximal to the NIRS optode and was used to occlude blood flow. NIRS data were collected at 10 Hz. Adipose tissue thickness at the site of measurement was assessed, as per the recommendations of Ferrari et al. (2011), using skinfold callipers. However, as demonstrated in Ryan et al. (2012), an ischaemic calibration eliminates any effect of adipose tissue thickness and scales the NIRS signals according to the maximal physiological range.

### Protocol

All visits followed a similar pattern of data acquisition to Pethick et al. (2019), though each visit was conducted at a different knee joint angle: either 30°, 60° or 90° of knee flexion. Visits began with the instrumentation of the participants and the (re-)establishment of the correct dynamometer seating position and supramaximal stimulation response. Participants then performed a series of brief (3 s) MVCs to establish the maximum torque at that joint angle. These MVCs were separated by a minimum of 60 s rest and continued until the peak torque in three consecutive contractions were within 5% of each other. Participants were given a countdown, followed by very strong verbal encouragement to maximise torque. The first MVC was used to establish the fresh maximal EMG signal, against which the subsequent EMG signals were normalised (“Data analysis”). The second and third MVCs were performed with femoral nerve stimulation delivered during and after the contraction. The stimulation during the contraction was delivered at a plateau in torque, to test the maximality of the contraction and provide the resting voluntary activation. The stimulation after the contraction was delivered at rest, 2 s after the contraction, to establish the fresh potentiated doublet torque. All subsequent contractions with femoral nerve stimulation were conducted in this manner.

Ten minutes after the establishment of maximal torque, the resting ${\text{m}\dot{\text{V}}\text{O}}_{{2}}$ of the vastus lateralis was assessed based on the decrease in muscle oxygenation which accompanies an arterial occlusion (Ryan et al. 2012, 2013). It must be noted that this method does not provide ${\text{m}\dot{\text{V}}\text{O}}_{{2}}$ in absolute terms; rather, it gives a measure of relative ${\text{m}\dot{\text{V}}\text{O}}_{{2}}$ in units of %·s^−1^, where % is an estimate of tissue O_2_ saturation. For this, a blood pressure cuff was rapidly inflated to a pressure of 300 mmHg using a Hokanson AG101 (D.E. Hokanson Inc., Bellevue, USA). Four resting measurements were made using 10 s of arterial occlusion, each separated by 60 s. The resting mV̇O_2_ was calculated using linear regression with the first 8 s of each occlusion (“Data analysis”). Participants then rested for 10 min before performing one of the experimental trials.

### Experimental trials

Participants performed a series of targeted intermittent isometric knee extension contractions at 25, 50, 75 and 100% MVC, to establish the relationship between complexity and contraction intensity at each joint angle. The target torques were determined from the highest instantaneous torque obtained during the pre-test MVCs. Participants performed three contractions at each intensity, with contractions held for 6 s and separated by 4 s rest. The intensities were performed in a randomised order, with 2 min rest between each intensity. Participants were instructed to match their instantaneous torque with a target bar superimposed on a display in front of them and were required to continue matching this torque for as much of the 6 s contraction as possible.

After participants had performed contractions at all four intensities, they rested for a further 10 min before performing an intermittent isometric fatigue test at 50% MVC. As with the targeted contractions, this torque was determined from the highest instantaneous torque obtained during the pre-test MVCs and the contractions were held for 6 s and separated by 4 s rest (Pethick et al. 2015, 2019). These contractions continued for 30 min or until task failure, whichever occurred sooner. Task failure was defined as the point at which the participants failed to reach the target torque on three consecutive occasions, despite strong verbal encouragement. Participants were not informed of the elapsed time during the trials but were informed of each “missed” contraction. Immediately at task failure, after the third missed contraction, participants were instructed to produce an MVC, which was accompanied by femoral nerve stimulation.

As in Pethick et al. (2019), after the fifth contraction of every minute of the fatigue test, ${\text{m}\dot{\text{V}}\text{O}}_{{2}}$ was assessed, instead of performing a targeted contraction. The blood pressure cuff was inflated to 300 mmHg for 5 s, with ${\text{m}\dot{\text{V}}\text{O}}_{{2}}$ calculated using linear regression over the course of this occlusion. This measure of ${\text{m}\dot{\text{V}}\text{O}}_{{2}}$ was performed instead of a targeted contraction. ${\text{m}\dot{\text{V}}\text{O}}_{{2}}$ was also assessed immediately prior to the MVC performed at task end/failure. Finally, 5 min after task end/failure, an ischaemia/hyperaemia calibration was performed to normalise the NIRS signals. The blood pressure cuff was inflated to 300 mmHg for 3–5 min (or until the NIRS signals plateaued). The plateau in HHb at the end of the occlusion was assumed to be the zero-point (the lowest functional level of HHb), with the peak response to hyperaemia upon cuff release being 100% oxygenation.

### Data acquisition and participant interface

Data acquisition was performed as described in Pethick et al. (2019). The isokinetic dynamometer, stimulator and EMG were connected via BNC cables to a Biopac MP150 (Biopac Systems Inc., California, USA) and a CED Micro 1401-3 (Cambridge Electronic Design, Cambridge, UK) interfaced with a personal computer. These data were sampled at 1 kHz and collected in Spike2 (Version 7; Cambridge Electronic Design, Cambridge, UK). The NIRS data were sampled at 10 Hz and collected in OxySoft (Artinis Medical Systems, Netherlands).

A chart containing the instantaneous torque was projected onto a screen placed ~ 1 m in front of the participant. A scale consisting of a thin line (1 mm thick) was superimposed on the torque chart and acted as a target, so that participants were able to match their instantaneous torque output to the target torque during each visit.

### Data analysis

All data were analysed using code written in MATLAB R2017a (The MathWorks, Massachusetts, USA). The data analysis focused on four specific areas: (1) basic measures of torque and EMG; (2) measures of central and peripheral fatigue; (3) the variability and complexity of torque output; and (4) measures of muscle oxygen consumption (${\text{m}\dot{\text{V}}\text{O}}_{{2}}$).

#### Torque and EMG

The mean and peak torque for each contraction in every trial were determined. The mean torque was calculated based on the steadiest 5 s of each contraction, with MATLAB code identifying the 5 s of each contraction with the lowest standard deviation (SD). The point of task failure in the fatigue test was determined as in Pethick et al. (2015). The mean torque produced during the first five contractions was calculated, with task failure deemed to occur when the mean torque recorded during three consecutive contractions was more than 5 N·m below the mean torque of the first five contractions, with the first of these contractions being considered the point of task failure.

The EMG outputs from the vastus lateralis and vastus medialis for each contraction were full-wave rectified during each 5 s window. The average rectified EMG (arEMG) was then calculated and normalised by expressing the arEMG as a fraction of the arEMG obtained during a 3 s MVC from the fresh muscle performed at the beginning of the trial.

#### Central and peripheral fatigue

Measures of central and peripheral fatigue were calculated from the stimuli delivered to the femoral nerve during and after the MVCs performed pre-test and at task failure. Peripheral fatigue was demonstrated by a fall in the potentiated doublet torque. Central fatigue was demonstrated by a decline in voluntary activation, as quantified using the twitch interpolation technique (Belanger and McComas 1981; Behm et al. 1996)

1 $${\text{Voluntary activation}} \left( \% \right) = 1 - ({\text{superimposed doublet}}/{\text{resting doublet}}) \times 100,$$

where the superimposed doublet was measured during the contraction of interest and the potentiated doublet was measured at rest 2 s after the contraction.

#### Variability and complexity

Measures of variability and complexity were calculated using the steadiest 5 s of each contraction (meaning 5000 data points were used), identified by MATLAB as the 5 s containing the lowest SD. The amount of variability in the torque output of each contraction was measured using the SD, which provides a measure of the absolute amount of variability in a time-series, and the coefficient of variation (CV), which provides a measure of the amount of variability in a time-series normalised to the mean of the time-series.

The temporal structure, or complexity, of torque output was examined using multiple time domain analyses, as recommended by Goldberger et al. (2002b). The regularity of torque output was determined using approximate entropy (ApEn; Pincus 1991) and the temporal fractal scaling of torque was estimated using the detrended fluctuation analysis (DFA; Peng et al. 1994) α scaling exponent. Sample entropy (Richman and Moorman 2000) was also calculated, but as shown in Pethick et al. (2015), this measure does not differ from ApEn when 5000 data points are used in the calculation. The calculations of ApEn and DFA are detailed in Pethick et al. (2015). In brief, ApEn was calculated with the template length, *m*, set at 2 and the tolerance, *r*, set at 10% of the SD of torque output, and DFA was calculated across time scales (57 boxes ranging from 1250 to 4 data points). In four trials, a degree of crossover (Hu et al. 2001) was identified in the log–log plot of fluctuation size versus box size (as shown by an *r* < 0.95). To account for this, a least-squares linear regression was used to fit two lines to the plot, and two α exponents were quantified. The second of these (α_2_, representing longer, physiologic, timescales) was used in the DFA α analysis (Pethick et al. 2019).

#### Muscle oxygen consumption

${\text{m}\dot{\text{V}}\text{O}}_{{2}}$ was determined as in Pethick et al. (2019) using the method of Ryan et al. (2012; 2013), in which relative ${\text{m}\dot{\text{V}}\text{O}}_{{2}}$ is calculated as the slope of the change in O_2_Hb and HHb during arterial occlusion using simple linear regression. The resting ${\text{m}\dot{\text{V}}\text{O}}_{{2}}$ was based on the first 8 s (80 data points) of a 10 s arterial occlusion, whilst the exercising ${\text{m}\dot{\text{V}}\text{O}}_{{2}}$ measurements were based on a 5 s arterial occlusion (50 data points).

The NIRS data were corrected for blood volume changes as described in Ryan et al. (2012; 2013), using custom-written MATLAB code. A blood volume correction factor (*β*) was calculated for each data point during the arterial occlusions

2 $$\beta \left(t\right)= \frac{\left|{O}_{2}Hb(t)\right|}{\left(\left|{O}_{2}Hb(t)\right|+ \left|HHb(t)\right|\right)},$$

where *β* is the blood volume correction factor, *t* is time, O_2_Hb is the oxygenated haemoglobin/myoglobin signal, and HHb is the deoxygenated haemoglobin/myoglobin signal. Each data point was corrected using its corresponding *β* according to Eqs. 3 and 4

3 $$O_{2} Hb_{c} \left( t \right) = O_{2} Hb\left( t \right) - \left[ {tHb\left( t \right) \times \left( {1 - \beta } \right)} \right],$$

4 $$HHb_{c} \left( t \right) = HHb\left( t \right) - \left[ {tHb\left( t \right) \times \beta } \right],$$

where O_2_Hb_c_ and HHb_c_ are the corrected oxygenated and deoxygenated haemoglobin/myoglobin signals, respectively; *t*Hb is the blood volume signal from the NIRS device; *β* is the blood volume correction factor; and *t* is time. The raw O_2_Hb signal in Eq. 3 is corrected by subtracting the proportion of the blood volume change attributed to O_2_Hb; whilst in Eq. 4, the raw HHb signal is corrected by subtracting the proportion of blood volume change attributed to HHb.

### Statistics

All data are presented as means ± SD. All data were tested for normality using the Shapiro–Wilk test. For the fatigue tests, two-way analysis of variance (ANOVAs) with repeated measures were used to test for differences between conditions and time points, and for a condition *x* time interaction for MVC torque, arEMG, potentiated doublet torque, voluntary activation, variability, complexity and ${\text{m}\dot{\text{V}}\text{O}}_{{2}}$. The variability, complexity, arEMG and ${\text{m}\dot{\text{V}}\text{O}}_{{2}}$ measures were analysed using means from the second minute, to account for the initial transient of the ${\dot{\text{V}}\text{O}}_{{2}}$ response (Burnley and Jones 2007) and the final minute before task end/failure. For the complexity–contraction intensity and variability–contraction intensity relationships, two-way ANOVAs with repeated measures were used to test for differences between conditions and contraction intensities, and for a condition *x* contraction intensity relationship for ApEn, DFA *α*, SD and CV. When main effects were observed, Bonferroni-adjusted 95% paired-samples confidence intervals were used to identify specific differences. The rates of change in all parameters during the fatigue test were analysed using Student’s paired-samples *t* tests. Correlations between rates of change in complexity and ${\text{m}\dot{\text{V}}\text{O}}_{{2}}$ were analysed using Pearson’s product–moment correlation (*r*) or, in the case of non-normally distributed data, Spearman’s rank-order correlation (*ρ*). Results were deemed statistically significant when *P* < 0.05.

## Results

### Preliminary measures

The contractile properties of the knee extensors are presented in Table 1. There was a significant effect of joint angle on pre-test MVC torque (*F* = 55.68, *P* < 0.001); with MVC torque lower at 30° compared to 60° (95% paired-samples confidence intervals [CIs]): – 125.4, – 66.7 N·m) and 90° (CIs: – 163.4, – 84.5 N·m). Similarly, there was a significant effect of joint angle on pre-test potentiated doublet torque (*F* = 20.36, *P* < 0.001); with potentiated doublet torque lower at 30° compared to 60° (CIs: – 35.5, – 4.6 N·m) and 90° (CIs: – 54.7, – 17.6 N·m). There was no effect of joint angle on pre-test voluntary activation.

**Table 1:** Voluntary torque, potentiated doublet torque, voluntary activation, EMG and ${\text{m}\dot{\text{V}}\text{O}}_{{2}}$ responses during contractions at 90°, 60° and 30°

Parameter	90°	60°	30°
Target torque, N·m	113.7 ± 29.4	100.0 ± 27.1	51.7 ± 14.9
Time to task end/failure, min	7.0 ± 4.1	13.4 ± 4.7^b^	27.9 ± 6.8^b,c^
Global fatigue			
Pre-exercise MVC, N·m	227.4 ± 58.7	200.0 ± 54.2	103.5 ± 29.8^b,c^
Peak MVC at task end/failure, N·m	124.4 ± 38.4^a^	111.8 ± 31.8^a^	82.9 ± 27.0^a^
Mean MVC at task end/failure, N·m	104.9 ± 31.3	105.2 ± 28.4	70.6 ± 22.8
% Change at task end/failure	45.5 ± 7.5	43.5 ± 8.8	19.5 ± 15.6^b,c^
∆MVC/∆t, N·m·min^−1^	–18.2 ± 8.6	–7.7 ± 4.2^b^	–1.3 ± 2.8^b,c^
Peripheral fatigue			
Pre-exercise doublet, N·m	89.6 ± 24.3	73.5 ± 31.4	50.4 ± 14.4^b,c^
Doublet at task end/failure, N·m	65.4 ± 19.0^a^	59.3 ± 18.8^a^	46.4 ± 15.6^a^
% Change at task end/failure	26.9 ± 10.1	19.1 ± 10.8	9.0 ± 7.6^b^
∆doublet/∆t, N·m·min^−1^	–4.3 ± 3.0	–1.2 ± 0.9^b^	–0.2 ± 0.4^b^
Central fatigue			
Pre-exercise VA, %	94.4 ± 1.4	91.2 ± 1.8	92.2 ± 4.0
VA at task end/failure, %	78.3 ± 6.5^a^	73.7 ± 8.5^a^	83.4 ± 11.9
% Change at task end/failure	17.1 ± 7.0	19.2 ± 9.3	9.4 ± 13.1
∆VA/∆t, %/min	–2.7 ± 1.4	–1.4 ± 0.7	–0.7 ± 1.8
Vastus lateralis EMG			
arEMG at task beginning, % MVC	62.4 ± 11.4	55.0 ± 8.9	45.5 ± 12.8^b^
arEMG at task end/failure, % MVC	75.4 ± 12.5^a^	81.3 ± 17.5^a^	51.0 ± 13.7^a^
∆arEMG/∆t, % MVC/min	3.9 ± 3.6	3.0 ± 2.6	0.3 ± 0.3^b,c^
Vastus medialis EMG			
arEMG at task beginning, % MVC	56.2 ± 12.4	53.0 ± 12.1	44.7 ± 10.4
arEMG at task end/failure, % MVC	74.2 ± 16.3^a^	76.4 ± 20.2^a^	50.5 ± 10.9
∆arEMG/∆t, % MVC/min	4.7 ± 2.9	2.3 ± 2.0	0.4 ± 0.9^b^
$${\text{m}\dot{\text{V}}\text{O}}_{{2}}$$			
${\text{m}\dot{\text{V}}\text{O}}_{{2}}$ at task beginning, %·s^−1^	2.3 ± 1.0	2.2 ± 1.0	1.8 ± 1.0
${\text{m}\dot{\text{V}}\text{O}}_{{2}}$ at task end/failure, %·s^−1^	3.3 ± 1.4^a^	3.2 ± 1.1^a^	2.1 ± 1.0^a^
Change in ${\text{m}\dot{\text{V}}\text{O}}_{{2}}$ at task end/failure	1.0 ± 0.5	1.1 ± 0.3	0.3 ± 0.2^b,c^
∆${\text{m}\dot{\text{V}}\text{O}}_{{2}}$/∆t, %·s^−1^·min^−1^	0.3 ± 0.1	0.1 ± 0.1^b^	0.01 ± 0.01^b,c^

Values are means ± SD. *MVC* maximal voluntary contraction, *VA* voluntary activation, *EMG* electromyogram, *arEMG* average rectified EMG of the vastus lateralis, ${\text{m}\dot{\text{V}}\text{O}}_{{2}}$ muscle oxygen consumption, ∆ change, *t* time. Task beginning values are values from 2 min into exercise, to account for primary amplitude of ${\dot{\text{V}}\text{O}}_{{2}}$ response. Symbols indicate a statistically significant difference compared to the following: ^a^pre-test/task beginning value, ^b^90°, ^c^60°

**Fig. 1:**
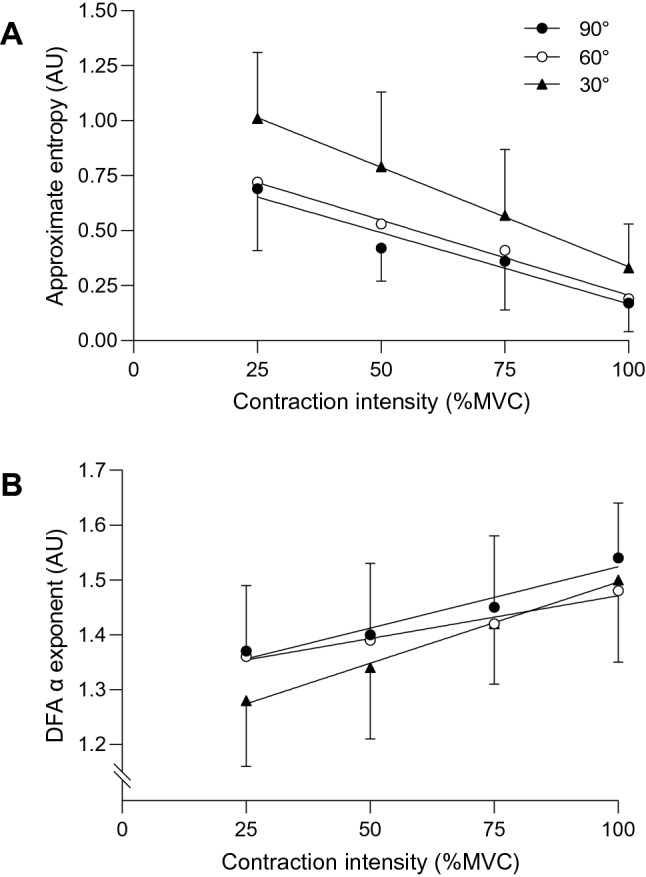
Relationship between contraction intensity and torque complexity at three muscle lengths. **A** Presents the relationship between approximate entropy and intensity, whilst **B** shows the relationship between the DFA α exponent and intensity across conditions

There was a significant effect of joint angle on the magnitude of variability, as measured by the SD (*F* = 2.91, *P* = 0.016), but not the CV (*F* = 3.50, *P* = 0.052). The SD was significantly lower at 30° compared to 90° at 50% (CIs: – 1.9, – 0.6 N·m) and 75% MVC (CIs: – 2.3, – 0.3 N·m), and was lower than at 60° during contractions at 100% MVC (CIs: – 5.3, – 0.9 N·m). The complexity–contraction intensity relationships are presented in Fig. 1. There was a significant effect of joint angle on complexity, as measured by ApEn (*F* = 7.69, *P* = 0.004), but not DFA α (*F* = 2.24, *P* = 0.135). ApEn decreased in a linear fashion as contraction intensity increased, regardless of joint angle (Fig. 1). ApEn was significantly greater at 30° compared to 90° during contractions at 50% (CIs: 0.09, 0.6) and 75% MVC (CIs: 0.04, 0.4), and was greater than at 60° during contractions at 25% (CIs: 0.02, 06) and 100% MVC (CIs: 0.0002, 0.3). There were no differences between 90° and 60° at any contraction intensity.

### Time to task failure, MVC torque and EMG

There was a significant effect of joint angle on time to task end/failure (*F* = 55.52, *P* < 0.001; Table 1). Time to task failure increased from 90° (7.0 ± 4.1 min), to 60° (13.4 ± 4.7 min), to 30° (27.9 ± 6.8 min), with 10 out of 11 participants completing 30 min of contractions at this angle. Time to task failure was significantly greater at 30° compared to 60° (CIs: 8.0, 20.0 min) and 90° (CIs: 14.7, 27.1 min), and greater at 60° compared to 90° (CIs: 0.7, 12.1 min).

Each condition resulted in significant decreases in MVC torque (*F* = 127.90, *P* < 0.001; Table 1). The MVC torque produced at task failure was not significantly different from the target torque at 60° (CIs:  – 1.8, 25.4 N·m) and 90° (CIs:  – 4.5, 28.5 N·m). In contrast, the MVC torque produced at task end/failure at 30° remained significantly greater than the target torque (CIs: 15.4, 46.9 N·m), indicating that these contractions ended with a substantial reserve in maximal torque. The magnitude of the decrease in MVC torque was significantly lower at 30° compared to 60° (CIs: – 42.3, – 5.6%) and 90° (CIs: – 42.8, – 9.2%). There was also a significant effect of joint angle on the rate of decrease in MVC torque (*F* = 35.13, *P* < 0.001; Table 1). The rate of decrease in MVC torque was significantly slower at 30° compared to 60° (CIs: – 9.3, – 3.4 N·m·min^−1^) and 90° (CIs: – 23.8, – 10.0 N·m·min^−1^), and at 60° compared to 90° (CIs: – 17.2, – 3.8 N·m·min^−1^).

**Fig. 2:**
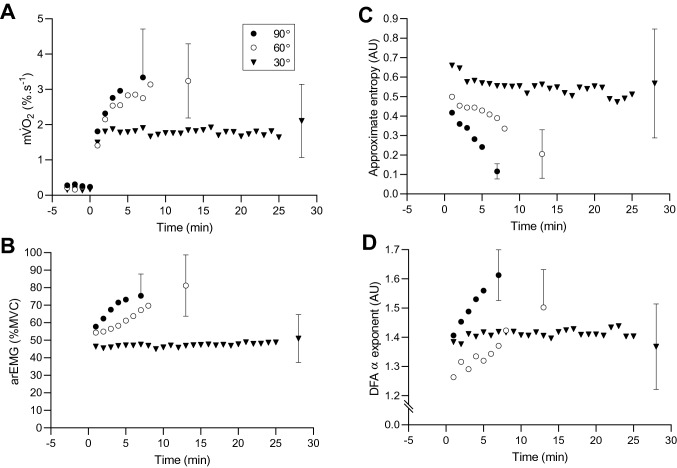
Muscle oxygen uptake (**A**), average rectified EMG (**B**) and complexity responses (ApEn, **C**; DFA α exponent, **D**) to contractions at three muscle lengths. Note the qualitatively similar patterns of response at each muscle length between variables, with task failure occurring later as muscle length is shortened from 90º to 30º. Also note the quasi-steady state responses in the 30º condition, suggesting that this was performed below the critical torque

Figure 2 shows the time course of ${\text{m}\dot{\text{V}}\text{O}}_{{2}}$, vastus lateralis arEMG and muscle torque complexity in each condition. The arEMG of the vastus lateralis significantly increased over time in each condition (*F* = 38.60, *P* = 0.001; Table 1). The arEMG of the vastus medialis significantly increased over time (*F* = 34.19, *P* < 0.001) at 60° (CIs: 2.2, 44.7%) and 90° (CIs: 8.3, 27.9%), but not at 30° (CIs: – 13.7, 2.2%). There were also significant effects of joint angle on the rate of increase in vastus lateralis (*F* = 7.67, *P* = 0.003; Table 1) and vastus medialis (*F* = 12.81, *P* < 0.001; Table 1) arEMG. The rate of increase in vastus lateralis arEMG was significantly slower at 30° compared to 60° (CIs: – 4.8, – 0.5%·min^−1^) and 90° (CIs: – 6.5, – 0.6%·min^−1^). The rate of increase in vastus medialis arEMG was significantly slower at 30° compared to 90° (CIs: – 6.5, – 2.0%·min^−1^).

### Peripheral and central fatigue

Each condition resulted in a significant reduction in potentiated doublet torque (*F* = 62.54, *P* < 0.001; Table 1), indicating the presence of peripheral fatigue. The magnitude of this reduction was, however, significantly lower at 30° compared to 90° (CIs: – 29.9, – 5.9 N·m). The rate of decrease in potentiated doublet torque was significantly slower at 30° compared to 90° (CIs: – 6.9, – 1.5 N·m·min^−1^; Table 1), and at 60° compared to 90° (CIs: – 5.7, – 0.6 N·m·min^−1^). Voluntary activation significantly decreased at 60° and 90° (*F* = 73.96, *P* < 0.001; Table 1), indicating the presence of central fatigue. There was no change in voluntary activation at 30° (CIs: – 2.6, 20.1%).

### Muscle oxygen consumption

Each condition resulted in a significant increase in ${\text{m}\dot{\text{V}}\text{O}}_{{2}}$ (*F* = 127.29, *P* < 0.001; Fig. 2; Table 1). The magnitude of this increase was, however, significantly lower at 30° compared to 60° (CIs: – 1.2, – 0.2%·s^−1^) and 90° (CIs: – 1.2, – 0.4%·s^−1^). There was also a significant effect of joint angle on the rate of change in ${\text{m}\dot{\text{V}}\text{O}}_{{2}}$ (*F* = 16.39, *P* < 0.001; Table 1). The rate of change in ${\text{m}\dot{\text{V}}\text{O}}_{{2}}$ was significantly slower at 30° compared to 60° (CIs: – 1.5, – 0.4%·s^−1^) and 90° (CIs: – 1.2, – 0.2%·s^−1^).

### Variability and complexity

**Fig. 3:**
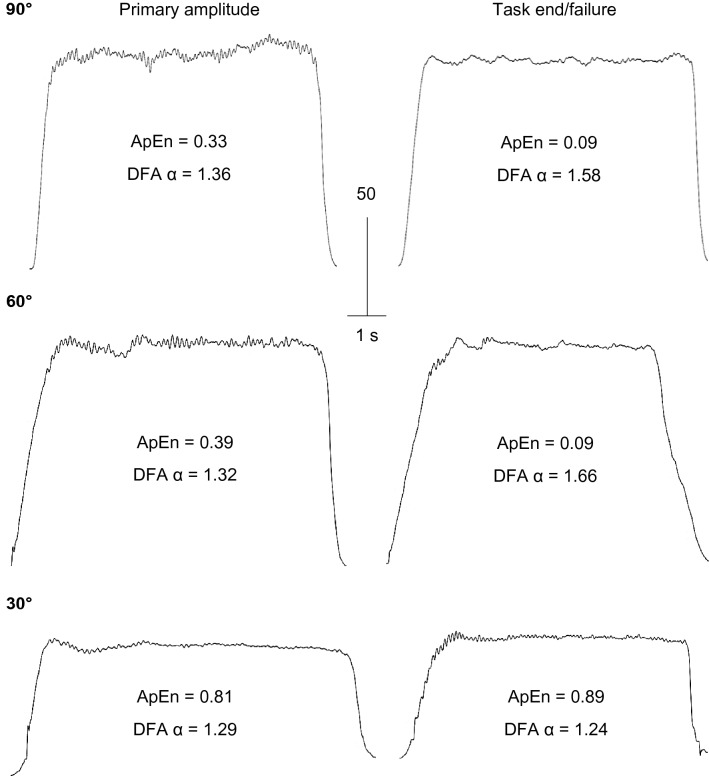
Raw muscle torque responses at the onset of contractions and at task failure or 30 min in each condition in a participant who did not reach task failure in the 30º condition. Note the absence of a change in complexity in the 30º condition, in contrast to the response at longer muscle lengths

The variability and complexity data from the fatigue test are presented in Table 2. Example contractions from the beginning and end of the fatigue test at each joint angle are presented in Fig. 3.

**Table 2:** Variability, complexity and fractal scaling responses during contractions at 90°, 60° and 30°

Parameter	90°	60°	30°
SD			
SD at task beginning, N·m	3.0 ± 1.1	2.4 ± 0.6	1.6 ± 0.5
SD at task failure, N·m	6.9 ± 3.2^a^	4.4 ± 2.1	1.7 ± 0.6
∆SD/∆t, N·m·min^−1^	1.2 ± 1.1	0.2 ± 0.06	0.03 ± 0.1^b^
CV			
CV at task beginning, %	2.7 ± 1.0	2.5 ± 0.6	3.3 ± 1.3
CV at task failure, %	6.8 ± 2.4^a^	4.7 ± 1.6	3.6 ± 0.9
ΔCV/Δt, %/min	1.1 ± 0.9	0.2 ± 0.02^c^	0.05 ± 0.1^b^
ApEn			
ApEn at task beginning	0.36 ± 0.14	0.45 ± 0.14	0.64 ± 0.32
ApEn at task failure	0.12 ± 0.04^a^	0.21 ± 0.12^a^	0.57 ± 0.28^b,c^
∆ApEn/∆t	− 0.07 ± 0.04	− 0.02 ± 0.01^b^	− 0.01 ± 0.03^b^
DFA α			
DFA α at task beginning	1.46 ± 0.09	1.32 ± 0.24	1.38 ± 0.13
DFA α at task failure	1.61 ± 0.09^a^	1.50 ± 0.13^a^	1.37 ± 0.15^b,c^
∆DFA α /∆t	0.05 ± 0.03	0.02 ± 0.03	0.001 ± 0.009^b^

Values are means ± SD. *SD* standard deviation, *CV* coefficient of variation, *ApEn* approximate entropy, *DFA α* detrended fluctuation analysis, ∆ change, *t* time. Task beginning values are values from 2 min into exercise, to account for primary amplitude of ${\dot{\text{V}}\text{O}}_{{2}}$ response. Symbols indicate a statistically significant difference compared to the following: ^a^value at task beginning, ^b^90°, ^c^60°

There was a significant effect of time on the magnitude of variability, as measured by both the SD (*F* = 41.83, *P* < 0.001) and CV (*F* = 58.52, *P* < 0.001). The SD (CIs: 1.5, 6.4 N·m) and CV (CIs: 2.1, 6.0%) increased with time at 90°, but not at 60° or 30°. The rates of increase in the SD and CV were significantly slower at 30° compared to 90° (CIs: SD, – 1.7, – 0.4 N·m·min^−1^; CV, – 1.9, – 0.2%·min^−1^), and the rate of increase in the CV was significantly slower at 60° compared to 90° (CIs: – 1.7, – 0.06%·min^−1^).

During the fatigue test, there was a significant effect of time on ApEn (*F* = 71.88, *P* = 0.001) and DFA α (*F* = 89.90, *P* < 0.001; Fig. 2). ApEn decreased significantly at 60° (CIs: – 0.3, – 0.2) and 90° (CIs: – 0.4, – 0.1), but not at 30°. At task end/failure, ApEn remained significantly higher at 30° compared to 60° (CIs: 0.6, 1.2) and 90° (CIs: 0.2, 0.7). Similarly, DFA α increased at 60° (CIs: 0.02, 0.4) and 90° (CIs: 0.09, 0.2), but not at 30°. At task end/failure, DFA α remained significantly lower at 30° compared to 60° (CIs: – 0.2, – 0.003) and 90° (CIs: – 0.3, – 0.1). There was a significant effect of joint angle on the rate of change in ApEn (*F* = 17.85, *P* < 0.001) and DFA α (*F* = 11.67, *P* < 0.001). The rate of decrease in ApEn was slower at 30° compared to 90° (CIs: – 0.09, – 0.03) and at 60° compared to 90° (CIs: – 0.08, – 0.008). The rate of increase in DFA *α* was slower at 30° compared to 90° (CIs: 0.01, 0.07).

### Correlations

**Fig. 4:**
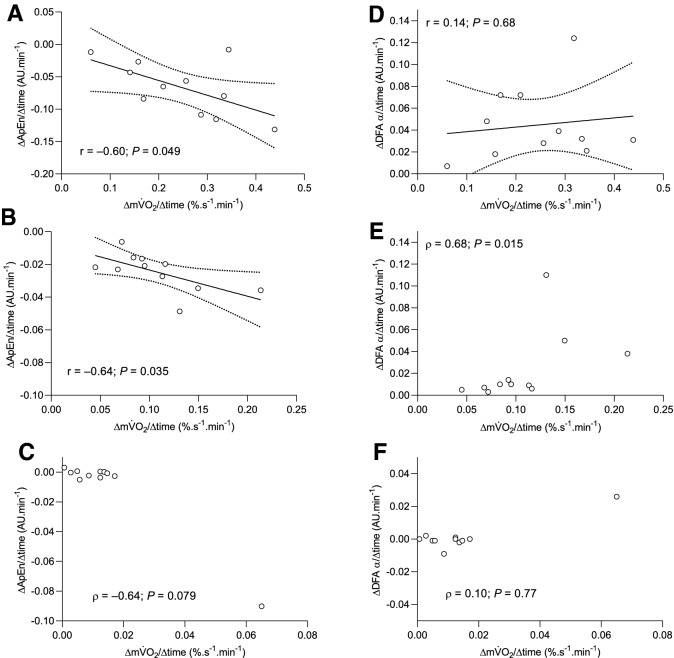
Correlations between complexity and muscle oxygen uptake at three different muscle lengths. **A**–**C** represent correlations between the change in ApEn and the change in ${\dot{\text{V}}\text{O}}_{{2}}$ at 90º, 60º and 30º, respectively and panels **D**–**F** represent the correlation between the change in DFA α and the change in muscle oxygen uptake at 90º, 60º and 30º, respectively. Note the significant but modest correlations in **A**, **B** and **E** (90º and 60º) and the lack of correlation at 30º (**C** and **F**)

Figure 4 shows the correlations between the rates of change in ${\text{m}\dot{\text{V}}\text{O}}_{{2}}$ and complexity during each condition. The rates of change in muscle torque complexity in the 60° condition, when quantified using DFA *α*, and in the 30° condition, when quantified using both ApEn and DFA *α*, were not normally distributed (Shapiro–Wilk test, *P* < 0.05). The Spearman’s rank correlation (*ρ*) was therefore used in these analyses. There were significant negative correlations between the rates of change in ApEn and ${\text{m}\dot{\text{V}}\text{O}}_{{2}}$ at 90° (*r* = –0.60, *P* = 0.049) and 60° (*r* =  – 0.64, *P* = 0.035), though not at 30° (*ρ* =  – 0.64, *P* = 0.079). There was a significant positive correlation between the rates of change in DFA *α* and ${\text{m}\dot{\text{V}}\text{O}}_{{2}}$ at 60° (*ρ* = 0.68, *P* = 0.015), though not at 90° (*r* = 0.14, *P* = 0.68) or 30° (*ρ* = 0.10, *P* = 0.77).

## Discussion

The primary aim of the present study was to investigate whether the inverse correlation between the fatigue-induced changes in muscle torque complexity and metabolic rate we have previously observed (Pethick et al. 2019) was conserved with changes in joint angle. Correlations between torque complexity and ${\text{m}\dot{\text{V}}\text{O}}_{{2}}$ were observed during contractions at 90° (for ApEn) and 60° (for ApEn and DFA α) but were absent during contractions at 30°. The lack of a correlation during the contractions at 30° was likely due to the low relative demands in this condition, as the kinetics of torque complexity and ${\text{m}\dot{\text{V}}\text{O}}_{{2}}$ (after the initial transient) were similar. A further aim of the study was to investigate the mechanistic basis for the greater endurance observed at more extended joint angles. This study demonstrated greater endurance and a significantly attenuated rate of change in ${\text{m}\dot{\text{V}}\text{O}}_{{2}}$ (and torque complexity) at more extended joint angles, providing support for the contention that muscle length-dependent changes in metabolic rate may play a role in the muscle length-endurance relationship.

### Muscle length–endurance relationship

In agreement with previous work, endurance time was inversely related to joint angle (Table 1; Ng et al. 1994; De Ruiter et al. 2005). The contractions performed at 90° and 60° resulted in inexorable changes in all variables until task failure, which occurred after 7.0 and 13.4 min, respectively. These responses are typical of exercise performed above the so-called critical torque, in the severe exercise intensity domain (Burnley and Jones 2007). In contrast, endurance during contractions at 30° was such that participants were able to continue exercise for 30 min (in 10 of 11 cases) without reaching task failure. Notably, these responses were more typical of those we have observed below the critical torque (Burnley et al. 2012; Pethick et al. 2016), in that increases in arEMG and ${\text{m}\dot{\text{V}}\text{O}}_{{2}}$ were of significantly smaller magnitude than those observed at 90° and 60°, peripheral fatigue developed only very slowly, and there was no change in muscle torque complexity. This invites the intriguing possibility that the greater endurance observed at 30° was because this exercise was, in fact, performed in the heavy exercise domain, *below* the critical torque.

The critical torque in the knee extensors has previously only been investigated during contractions at 90°, where it has been demonstrated to occur at ~ 30% MVC (Burnley 2009; Pethick et al. 2016). If that figure were applied to the present data, critical torque at 30° would equate to 30.3 ± 2.9 N·m, considerably lower than the target torque of 51.7 ± 4.8 N·m. As such, it would have been expected that task failure would have occurred. That it did not in the majority of cases suggests that critical torque occurs at a greater fraction of MVC at shorter muscle lengths. Further support for the contention that the contractions at 30° were performed below the critical torque comes from previous observations that the target torque for contractions at 30° had to be raised from 50 to ~ 70% MVC to produce similar responses to contractions at 90° (De Ruiter et al. 2005; Kooistra et al. 2005). Therefore, it appears that shortening muscle length reduces the force-generating capacity of the muscle to a considerably greater degree than it reduces the muscle’s endurance capacity.

The rate of increase in ${\text{m}\dot{\text{V}}\text{O}}_{{2}}$ was significantly slower at more extended joint angles (Table 1), providing support for the contention that lower metabolic rate may contribute to the increased time to task failure seen at more extended joint angles (De Ruiter et al. 2005; Kooistra et al. 2006). The mechanism(s) responsible for this slower increase in metabolic rate remains obscure, though could be viewed in the following way: maintaining torque output in the face of developing fatigue requires the recruitment of additional motor units, which can be reflected by a greater arEMG amplitude (Moritani et al. 1986; Krogh-Lund and Jorgensen, 1992). The recruitment of additional motor units is temporally associated with increases in ${\text{m}\dot{\text{V}}\text{O}}_{{2}}$ (beyond the initial transient; Poole et al. 1991; Krustrup et al. 2004). The rate of decrease in MVC torque was slower at 30°, due to the relatively low demands of the task and thus the recruitment of fewer additional motor units in order to maintain task demands. This may have been reflected in the slower rate of increase in vastus lateralis (and medialis) arEMG and ${\text{m}\dot{\text{V}}\text{O}}_{{2}}$. In support of this contention, Weir et al. (2000) concluded that greater changes in the rectified EMG and mechanomyogram during contractions at long compared to short muscle lengths were due to a greater rate of motor unit recruitment.

### Relationship between muscle length, metabolic rate and torque complexity

This study demonstrated that the previously observed fatigue-induced loss of muscle torque complexity (Pethick et al. 2015, 2016), measured by decreased ApEn (indicating increased regularity) and increased DFA *α* (indicating increasingly Brownian fluctuations in torque) was also evident during contractions at intermediate joint angles, i.e., 60° of knee flexion (Fig. 2; Table 2). In contrast, at the more extended joint angle of 30°, where most participants were able to continue exercise for 30 min without reaching task failure, torque complexity did not decrease. This would suggest that, based on the purported significance of muscle torque complexity (Vaillancourt and Newell 2003; Pethick et al. 2016), the adaptability of motor output was maintained and that the contractions ended with a significant reserve in exercise capacity. This was confirmed by the observation that MVC torque at task end remained significantly greater than the target torque, in contrast to the other two conditions.

The rate of the fatigue-induced loss of muscle torque complexity was significantly slowed as joint angle decreased and time to task failure/end increased (Table 2). The rate of change in muscle torque complexity exhibited a modest negative correlation with the rate of change in ${\text{m}\dot{\text{V}}\text{O}}_{{2}}$ during contractions at 90° (ApEn only) and 60° (ApEn and DFA *α*; Fig. 4). This is in line with Seely and Macklem’s (2012) hypothesised inverse relationship between complexity and metabolic rate, along with our own previous findings (Pethick et al. 2019). During the contractions at 30°, no significant correlations were evident, though the kinetics of muscle torque complexity and ${\text{m}\dot{\text{V}}\text{O}}_{{2}}$ were qualitatively similar (Fig. 2). The significant correlations observed in our present and previous work (Pethick et al. 2019) were only ever of a modest nature (*r* =  ~ 0.6), suggesting that no more than 35–40% of the variance in muscle torque complexity could be explained by the increase in metabolic rate. Changes in muscle torque complexity and ${\text{m}\dot{\text{V}}\text{O}}_{{2}}$ do appear to be qualitatively similar at all joint angles (Fig. 2) and it is likely that the two are related. The modest strength of the relationship indicates that it may be mediated by a co-variate which causes both to change, with the most likely candidate for this co-variate being motor unit behaviour (Pethick et al. 2019).

The development of neuromuscular fatigue necessitates the recruitment of additional motor units to maintain torque output. This activation of a greater proportion of the motor unit pool serves to increase ${\text{m}\dot{\text{V}}\text{O}}_{{2}}$ and diminish the adaptive capacity of the muscle, reflected in the loss of muscle torque complexity (Pethick et al. 2019). We have suggested that a specific aspect of motor unit behaviour, namely common synaptic input, is responsible for the loss of muscle torque complexity (Pethick et al. 2018). Common synaptic input has been proposed as the major determinant of the magnitude of force fluctuations (Farina and Negro 2015), based on the close relationship between the cumulative motor unit spike train and muscle force output (Negro et al. 2009; Thompson et al. 2018). Moreover, common synaptic input, and its necessary consequence motor unit synchronisation, has been demonstrated to increase as neuromuscular fatigue develops (Castronovo et al. 2015). Motor unit synchronisation has been implicated in the fatigue-induced loss of complexity in EMG output (Beretta-Piccoli et al. 2015) and an age-induced loss of complexity in postural tremor (Sturman et al. 2005), and has been demonstrated, in a simulation study, to decrease force steadiness (Yao et al. 2000). Whilst such findings suggest a role for common synaptic input and motor unit synchronisation in the fatigue-induced loss of muscle torque complexity, direct measurement of motor unit spike trains, using either high-density or intramuscular EMG, is necessary to confirm this.

We have previously asserted that peripheral fatigue is also a major contributor to the loss of muscle torque complexity; acting, at the very least, as a pre-requisite for the central adjustments that act on the motor unit pool and themselves are responsible for the loss of complexity (Pethick et al. 2016, 2018). In the present study, a modest degree of peripheral fatigue developed during the contractions at 30° (Table 1) without a concomitant decrease in muscle torque complexity (Table 2). This suggests that the magnitude of peripheral fatigue incurred at 30°, whilst significant, was not sufficient to initiate a loss of complexity. We have observed similar small, but significant, peripheral fatigue and no change in muscle torque complexity during contractions performed below the critical torque (Pethick et al. 2016). These results suggest that the neuromuscular system can develop a modest degree of peripheral fatigue whilst maintaining the adaptability of motor output. Thus, the mere presence of peripheral fatigue is not enough to initiate the chain of events that affect the motor pool and lead to the loss of complexity; rather, a certain threshold must be exceeded in order for complexity to be perturbed.

### Complexity–contraction relationship

Our previous work has demonstrated a linear relationship between complexity and contraction intensity during knee extension contractions at 90° (Pethick et al. 2016). However, the only previous study to examine the complexity–contraction intensity relationship at different joint angles found a significant quadratic (shallow *U*-shaped) relationship during knee extension contractions at 40° and no relationship at 100° for ApEn (Ofori et al. 2018). The present results demonstrated that ApEn decreased in a linear fashion as contraction intensity increased for all joint angles tested (Fig. 1). Moreover, ApEn was consistently greater at 30°, suggesting, in line with the purported significance of complexity, a greater adaptive capacity (Vaillancourt and Newell 2003). The decrease in muscle torque complexity with increasing contraction intensity at each joint angle most likely relates to common synaptic input to muscle, which increases as the net excitatory input to muscle increases (Castronovo et al. 2015). The greater complexity at shorter muscle lengths could also be related to differences in excitatory input. Becker and Awiszus (2001) postulated that at more extended knee joint angles, there is less stretch is placed on muscle spindles (Ia afferents) and that, consequently, there is less excitatory drive to the motoneuron pool.

### Limitations

The present study was subject to some limitations. Differences in the kinetics of [HHb] have been observed between the vastus lateralis and rectus femoris (Koga et al. 2017) and along the length of the vastus lateralis (Koga et al. 2007). Our measure of ${\text{m}\dot{\text{V}}\text{O}}_{{2}}$ is, therefore, limited not only to the vastus lateralis but a specific region of the vastus lateralis, namely a small and superficial area of muscle under the optode, which was placed at the largest circumference of the thigh. Similarly, a recent review has highlighted that the accurate assessment of muscle excitation requires measurement from multiple surface electromyograms, to detect spatial heterogeneity along the muscle (Vieira and Botter 2021). The limitations in the location of our sampling may be of further importance due to differences in the relative contributions to torque of the vastus lateralis, vastus medialis and rectus femoris with changes in joint angle (Pincivero et al. 2004). Nevertheless, the differences in the behaviour of muscle torque complexity, ${\text{m}\dot{\text{V}}\text{O}}_{{2}}$ and arEMG between joint angles are much larger than regional variations typically observed across the quadriceps femoris.

### Conclusions

In summary, the present study has provided further evidence that muscle torque complexity and metabolic rate are related and demonstrated that joint angle has a significant influence on the fatigue-induced loss of muscle torque complexity. Specifically, more extended joint angles slowed the rate at which muscle torque complexity decreased, concomitant to a slowing of the increase in ${\text{m}\dot{\text{V}}\text{O}}_{{2}}$ and the development of neuromuscular fatigue. During contractions performed at knee joint angles of 90° and 60° of flexion, the rates of change in muscle torque complexity and ${\text{m}\dot{\text{V}}\text{O}}_{{2}}$ exhibited a modest negative correlation. Though no correlations were observed during contractions at 30° of flexion, the temporal profiles of the rates of change in muscle torque complexity and ${\text{m}\dot{\text{V}}\text{O}}_{{2}}$ were, nevertheless, qualitatively similar. The ${\text{m}\dot{\text{V}}\text{O}}_{{2}}$ and fatigue response profiles in the 30° condition suggested that it was performed in the heavy intensity domain. The modest nature of the relationship between muscle torque complexity and metabolic rate suggests that these variables are related, but that a common co-variate mediates the behaviour of both.

## Data Availability

Not applicable.

## Abbreviations

ApEn: Approximate entropy
DFA: Detrended fluctuation analysis
MVC: Maximal voluntary contraction
